# Can healthcare utilization data reliably capture cases of chronic respiratory diseases? a cross-sectional investigation in Italy

**DOI:** 10.1186/s12890-016-0362-6

**Published:** 2017-01-19

**Authors:** A Biffi, R Comoretto, A Arfè, L Scotti, L Merlino, A Vaghi, A Pesci, R de Marco, G Corrao, on behalf of the “CRD Real-World Evidence” scientific board, Ovidio Brignoli, Isa Cerveri, Giovanni Corrao, Eugenio Guffanti, Adriano Vaghi, Marco Villa, Roberto de Marco

**Affiliations:** 1 Laboratory of Healthcare Research and Pharmacoepidemiology, Department of Statistics and Quantitative Methods, University of Milano-Bicocca, Via Bicocca degli Arcimboldi, 8, Edificio U7, 20126 Milan, Italy; 2 Department of Decision Sciences, Bocconi University, Milan, Italy; 3 Operative Unit of Territorial Health Services, Region Lombardia, Milan, Italy; 4 Division of Pneumology, “Guido Salvini” Hospital, Garbagnate Milanese, Italy; 5 Department of Medicine and Surgery, University of Milano-Bicocca, Milan, Italy; 6 Division of Pneumology, “San Gerardo” Hospital, Monza, Italy; 7 Unit of Epidemiology and Medical Statistics, University of Verona, Verona, Italy

**Keywords:** Prevalence, Asthma, Chronic obstructive pulmonary disease, Healthcare utilization database, Algorithms

## Abstract

**Background:**

Healthcare utilization data are increasingly used for chronic disease surveillance. Nevertheless, no standard criteria for estimating prevalence of high-impact diseases, such as chronic obstructive pulmonary disease (COPD) and asthma, are available. In this study an algorithm for recognizing COPD/asthma cases from HCU data is developed and implemented in the HCU databases of the Italian Lombardy Region (about 10 million residents). The impact of diagnostic misclassification for reliably estimating prevalence was also assessed.

**Methods:**

Disease-specificdrug codes, hospital discharges together with co-payment exemptions when available, and a combination of them according with patient’s age, were used to create the proposed algorithm. Identified cases were considered for prevalence estimation. An external validation study was also performed in order to evaluate systematic uncertainty of prevalence estimates.

**Results:**

Raw prevalence of COPD and asthma in 2010 was 3.6 and 3.3% respectively. According to external validation, sensitivity values were 53% for COPD and 39% for asthma. Adjusted prevalence estimates were respectively 6.8 and 8.5% for COPD (among person aged 40 years or older) and asthma (among person aged 40 years or younger).

**Conclusions:**

COPD and asthma prevalence may be estimated from HCU data, albeit with high systematic uncertainty. Validation is recommended in this setting.

**Electronic supplementary material:**

The online version of this article (doi:10.1186/s12890-016-0362-6) contains supplementary material, which is available to authorized users.

## Background

Chronic obstructive pulmonary disease (COPD) and asthma are two of the most common chronic respiratory diseases (CRDs). CRDs have a large impact on public health due to both their high prevalence and related morbidity and mortality and their substantial socio-economic costs [1–3].

Assessing the burden of CRDs through valid and updated estimates of their prevalence, may help healthcare decision makers in guiding public health policy, even though such estimates are not easy to obtain.

Since patients affected by CRDs plausibly make use of healthcare services during the course of their disease, healthcare utilization (HCU) databases are frequently considered as useful data sources to capture CRD cases and estimate their prevalence in large unselected populations [4–7]. To this aim, investigators typically use ad-hoc algorithms based on the use of healthcare services, such as drug dispensations or hospital admissions. Clearly, such algorithms may be characterized by different operating characteristics, but validated standards are currently unavailable in this setting.

To assess the reliability of the algorithms in capturing patients affected by COPD and asthma, we conducted a cross-sectional study based on the HCU databases of the Italian Lombardy Region. In this study, we i) employed several algorithms to detect COPD and asthma cases from HCU data, ii) assessed the agreement between them, iii) evaluated the impact of misclassification on the prevalence estimates of COPD and asthma and iv) compared our prevalence with those available from the scientific literature.

## Methods

### Data source

The data used for this study were retrieved from the HCU databases of Lombardy, a Region of Italy accounting for about 16% (almost 10,000,000) of the national population. In Italy, the entire population benefits from healthcare assistance provided by the National Health Service (NHS), which in Lombardy has been associated since 1997 with an automated system of databases. Among others, these include: 1) an archive of NHS beneficiaries (practically the whole resident population), reporting demographic and administrative data; 2) a hospital discharge database, reporting all discharge diagnoses released from public or private hospitals; 3) an outpatient drug prescriptions database, reporting all dispensations of drugs reimbursable by the NHS; and 4) an archive of co-payment exemptions reporting information on all beneficiaries of co-payment exemptions granted for selected chronic diseases. For each patient, we linked these databases via a single anonymous identification code in full preservation of individuals’ privacy [8].

### Algorithms for case detection and prevalence estimation

The target population consisted in all beneficiaries of NHS assistance, residing in Lombardy Region in 2010.

Three algorithms were considered for detecting patients suffering of COPD and asthma from HCU databases. The first one, denoted as reference algorithm, was based on expert opinions of the scientific board of CRACK-CRD program, composed by general practitioner, lung specialists and epidemiologists. This algorithm was obtained combining age (<40 year for asthma and ≥40 years for BPCO) and use of healthcare services considering the hospital discharges, drug dispensations and co-payment exemptions (asthma only) recorded in the databases during 2010 for capturing the target diseases (Table 1).

**Table 1:** Reference algorithm used to capture COPD and asthma cases among the beneficiaries of the Regional Health Service. Lombardy, Italy

COPD	Asthma
≥40 years of age *And at least one of the following criteria* ^a^ *:* ≥1 hospitalizations for COPD ≥2 prescr/year of tiotropium or LABAs (in monotherapy), LABA/ICS in fixed combinations, SABAs ≥2 prescr/year of ICS in addition to ≥2 prescr/year of ipratropium or oxitropium ≥2 prescr/year of theophylline and ≥2 prescr/year of any of the following drugs:beta-agonists, ipratropium, oxitropium, inhaled corticosteroids	<40 years of age *And at least one of the following criteria* ^a^ *:* Benefitted of exemption for asthma ≥1 hospitalizations for asthma ≥2 prescr/year of any of the following drugs: anti-leukotrienes, chromones, ≥2 prescr/year of any of the following drugs: SABAs, LABA/ICS fixed combinations (only 0–19 age) ≥2 prescr/year of ICS in addition to ≥1 prescr/year of SABAs ≥2 prescr/year of ICS in addition to ≥2 prescr/year of ipratropium, oxitropium or theophylline (only 0–19 age) ≥1 prescr/year of antibiotics (ATC: J01; only 20–39 age) in addition to at least one of the following criteria: ≥2 prescr/year of theophylline and ≥2 prescr/year of any of the following drugs: beta-agonists, ipratropium, oxitropium, inhaled corticosteroids ≥2 prescr/year of SABAs or LABA/ICS fixed combinations ≥2 prescr/year. of ICS in addition to ≥2 prescr/year of ipratropium or oxitropium

To be recognized as affected by COPD/asthma, an individual must satisfy at least one of the listed criteria evaluated in 2010

*Abbreviations*: *prescr/yr.* prescription per year

^a^The specific ATC, ICD-9 CM and exemption codes used to identify asthma and COPD cases are reported in Additional file 1: Table S1

Starting from the criteria proposed by Anecchino et al. [9] for COPD, and Bianchi et al. [10] for asthma, two comparison algorithms were also implemented taking into account for case definition age (<40 year for asthma and ≥40 years for BPCO) and drug dispensations only. The drugs considered in these algorithms are those included in the reference algorithm. In particular, patients were identified as cases if they received at least one (permissive algorithm), or ≥2 dispensations (restrictive algorithm) of the considered medicaments during 2010 (Table 2).

**Table 2:** Comparison algorithms used to capture COPD and asthma cases among the beneficiaries of the Regional Health Service. Lombardy, Italy

COPD	Asthma
Permissive algorithm	
≥40 years of age *And at least one of the following criteria* ^a^ *:* ≥1 prescr/year of one of the following drugs: SABA, SAMA, LAMA, LABA, LABA/ICS, ICS	<40 years of age *And at least one of the following criteria* ^a^ *:* ≥1 prescr/year of one of the following drugs: SABA, LABA, LABA/ICS, ICS, chromones, anticholinergics, theophylline ≥1 prescr/year of anti-leukotrienes and ≥1 prescr/year of any other drug for chronic respiratory diseases ≥1 prescr/year of oral steroids and ≥1 prescr/year of any other drug for chronic respiratory diseases
Restrictive algorithm	
≥40 years of age *And at least one of the following criteria* ^a^ *:* ≥2 prescr/year of one of the following drugs: SABA, SAMA, LAMA, LABA, LABA/ICS, ICS	<40 years of age *And at least one of the following criteria* ^a^ *:* ≥2 prescr/year of one of the following drugs: SABA, LABA, LABA/ICS, ICS, chromones, anticholinergics, theophylline ≥2 prescr/year of anti-leukotrienes and ≥2 prescr/year of any other drug for chronic respiratory diseases ≥2 prescr/year of oral steroids and ≥2 prescr/year of any other drug for chronic respiratory diseases

To be recognized as affected by COPD/asthma, an individual must satisfy at least one of the listed criteria evaluated in 2010

*Abbreviations*: *prescr/year.* prescription per year

^a^The specific ATC codes used to identify asthma and COPD cases are reported in Additional file 1: Table S1

The specific codes used to identify the asthma and COPD cases in terms of drug prescriptions (ATC codes) [9, 10], diagnosis at discharge (ICD-9 CM codes) [11] for asthma and COPD and exemptions for asthma [12] for the three algorithms are listed in the Additional file 1: Table S1.

Cases detected from each of these algorithms following the criteria reported in Tables 1 and 2 were considered to estimate the raw prevalence of asthma and COPD considering all beneficiaries of NHS assistance during 2010 as reference population.

### Assessing between algorithms agreement

The between algorithms agreement in detecting patients suffering of COPD and asthma was measured by means of Cohen’s Kappa (K) index [13]. Following Landis & Koch [14], values K ≥0.80 were considered as representing optimal agreement.

### External validation and accounting for misclassification

The algorithms’ validation was performed involving data retrieved from a network of about 50 general practitioners (GPs) from Lombardy Region participating in the network on voluntary basis. In particular, the GPs identified among their patients those with a diagnosis of COPD or asthma based on standard practice criteria including the evaluation of the manifestation of the disease, patients characteristics such history of chronic or recurrent cough, sputum, wheezing or shortness of breath or based on the diagnosis of COPD or asthma made by a lung specialists or other specialized doctor. The information of these patients were then, reported to us. Assuming that the GP’s diagnosis were errors free, the proportion of individuals detected by a given algorithm as suffering from COPD or asthma among those reported by GPs defines the sensitivity (SE) of that algorithm.

The method proposed by Rogan & Gladen [15] was used to account for diagnostic misclassification. In particular, assuming 100% specificity, the adjusted prevalence was calculated by the ratio between raw prevalence and SE.

### Comparison with the literature

We carried out a MEDLINE / GOOGLE SCHOLAR search of studies published from 2005 to 2013 reporting prevalence of COPD or asthma in Italy. The studies were classified according to the data source. In particular, we included studies based on HCU databases, network of GPs and population-based survey. The prevalence reported by each individual study was compared to that obtained applying our reference algorithm to Lombardy’s HCU databases considering the same calendar years and age range of the considered study. Both raw and adjusted prevalence derived from Lombardy HCU databases were reported.

Statistical analyses were performed using SAS (v9.3; SAS Institute, Cary, North Carolina, USA).

## Results

In 2010 there were 10,172,161 NHS beneficiaries in Lombardy (43% with age <40 years).

The number COPD and asthma cases detected in this population varied substantially depending on the considered algorithm (Table 3).

**Table 3:** Evaluation of the agreement between reference and comparison algorithms and detection of their sensitivity. Lombardy, Italy, 2010

Algorithm	# patients (prevalence %)	Cohen’s Kappa ^a^	Sensitivity ^b^
COPD (patients aged 40 years or older)			
Reference algorithm ^c^	206,732 (3.6%)	-	52.6%
Permissive algorithm ^d^	545,520 (9.4%)	0.46	72.9%
Restrictive algorithm ^d^	217,095 (3.8%)	0.75	51.0%
Asthma (patients aged 40 years or younger)			
Reference algorithm ^c^	143,171 (3.3%)	-	38.8%
Permissive algorithm ^d^	440,130 (10.1%)	0.35	63.4%
Restrictive algorithm ^d^	142,482 (3.3%)	0.61	30.6%

^a^Agreement between reference and comparison (permissive and restrictive) algorithms; ^b^ Obtained from external validation data; ^c^ Please see Table 1 for details on reference algorithm; ^d^ Please see Table 2 for details on permissive and restrictive algorithms

The majority of patients with asthma were detected by the reference algorithm through prescriptions (74%) and exemptions (16%). Only the 1% of cases was identified by hospitalization only. Even for COPD the largest number of cases was identified through prescriptions (84%), the 7% by hospitalization only and the 9% using both sources.

Apparently, there were not substantial differences in terms of prevalence between the reference and restrictive algorithms, in fact the prevalence estimates were 3.3% for asthma considering both algorithms and 3.6 or 3.8% for COPD respectively for the reference and restrictive algorithms while the permissive algorithm reports much higher estimates. Moderate and fair agreement according to Landis and Koch scale was observed between reference and the permissive comparison algorithm respectively for COPD (Kappa index = 0.46) and asthma (Kappa index = 0.35) suggesting that the two algorithms often detected different patients. A substantial agreement was instead observed for the restrictive algorithm for both the respiratory diseases investigated. Moreover, both reference and restrictive algorithms detected just over half of the patients suffering of COPD (being the corresponding sensitivity estimates 53 and 51%) and almost a third of those suffering of asthma (being the corresponding sensitivity estimates 39 and 31%). As expected, a higher number of cases was detected from the permissive algorithm, but the strong disagreement with the reference one suggests that most of them were false positives or, conversely, that the reference algorithm is unable to detect all potential asthma and COPD cases due to its low sensitivity.

In Table 4 are reported the age and gender specific asthma and COPD distribution and prevalence according to the different algorithms implemented.

**Table 4:** Age and gender specific asthma and COPD distribution and prevalence according to the different algorithms implemented

	Age	Males (%)	Prevalence %	Females (%)	Prevalence %
COPD (patients aged 40 years or older)					
Reference algorithm	40–59	24,622 (23.1)	1.6	27,082(27.0)	8.3
	60–79	59,746 (56.1)	6.0	49,430(49.3)	11.3
	80+	22,120 (20.8)	10.6	23,733(23.7)	11.7
	Total	106,488	3.9	100,244	9.9
Permissive algorithm	40–59	90,918 (37.6)	6.0	123,519 (40.7)	8.3
	60–79	113,182 (46.8)	11.3	129,519 (42.7)	11.3
	80+	37,803 (15.6)	18.0	50,579 (16.7)	11.7
	Total	241,903	8.9	303,617	9.9
Restrictive algorithm	40–59	27,154 (25.4)	1.8	32,538 (29.5)	2.2
	60–79	56,286 (52.6)	5.6	52,108 (47.4)	4.6
	80+	23,611 (22.0)	11.3	25,398 (23.1)	5.9
	Total	107,051	3.9	110,044	3.6
Asthma (patients aged 40 years or younger)					
Reference algorithm	0–19	18,770 (48.6)	1.9	40,489 (38.7)	4.4
	20–39	19,844 (51.4)	1.6	64,069 (61.3)	5.3
	Total	38,613	1.7	104,558	4.9
Permissive algorithm	0–19	169,642 (73.5)	17.2	132,105 (63.1)	14.3
	20–39	61,046 (26.5)	4.9	77,337 (36.9)	6.4
	Total	230,688	10.4	209,442	9.9
Restrictive algorithm	0–19	57,395 (71.7)	5.8	38,572 (61.8)	4.2
	20–39	22,698 (28.3)	1.8	23,817 (38.2)	2.0
	Total	80,093	3.6	62,389	2.9

Prevalence of COPD seems to increase with age in both males and females according to all algorithms. Regarding gender differences, considering the reference algorithm, the prevalence seems higher in females than in males while no strong differences were observed in the gender-specific estimates obtained with the other algorithms. Regarding asthma, the estimates are higher in men than in women and in particular in the age class 0–19 years.

In Table 5 the prevalence estimates reported from selected Italian studies are compared [6, 9, 10, 16–24] with those obtained from our reference algorithm.

**Table 5:** COPD and asthma prevalence reported from selected cross-sectional Italian investigations compared with raw and adjusted estimates obtained by our reference algorithm

Data source	First author, publication year, [reference]	Year	Age (years)	Prevalence (%)		
				Original study	Reference algorithm, row estimate	Reference algorithm, misclassification adjusted estimate
COPD						
HCU	Anecchino, 2007 [9]	2004	≥45	3.6	3.8	7.2
	Faustini, 2008 [17]	2004	≥35	3.3	2.9	5.5
	Faustini, 2012 [16]	2006	≥35	5.7	3.0	5.7
	Gini, 2013 [6]	2009	≥16	4.7	2.5	4.7
GPs	Cazzola, 2011 [18]	2009	≥15	2.8	2.3	4.3
Surveys	ISTAT, 2008 [23]	2005	All ages	4.5	1.9	3.6
	de Marco, 2013 [20]	2007	20–84	7.2	2.3	4.3
Asthma						
HCU	Bianchi, 2011 [10]	2005	6–17	3.5	3.1	7.9
GPs	Cazzola, 2011 [18]	2009	≥15	6.1	1.5	3.8
Surveys	ISTAT, 2008 [23]	2005	All ages	3.5	1.8	4.6
	Demoly, 2009 [21]	2006	≥18	4.7	1.3	3.3
	To, 2012 [24]	2002–2003	≥18	6.1	1.6	4.1
	Jarvis, 2012 [22]	2008–2009	15–74	10.7	1.8	4.6
	de Marco, 2012 [19]	2007–2010	20–44	6.6	2.4	6.1
	de Marco, 2013 [20]	2007	20–84	8.8	1.4	3.6

Studies based on HCU data generally reported COPD and asthma prevalence very close to that obtained from our reference algorithm (raw prevalence). Similar COPD prevalence was also obtained from the unique study based on GPs data and our reference algorithm. In all the other cases (i.e., surveys reporting COPD or asthma prevalence and GPs-based studies reporting asthma prevalence) much lower prevalence was obtained from our reference algorithm respect to the original reporting. As expected, closest estimates were obtained accounting for diagnostic misclassification, although original prevalence based on surveys almost always showed higher values. Finally, it should be emphasized that original estimates were more heterogeneous than those based on our algorithm. In fact, estimates ranged from 2.8 to 7.2% and from 3.6 to 7.2% according to original and algorithm-based COPD prevalence, and from 3.5 to 10.7% and from 3.3 to 7.9% according to original and algorithm-based asthma prevalence.

## Discussion

An algorithm for detecting patients suffering from COPD and asthma from HCU databases was applied to the population of the Italian Region of Lombardy in the year 2010. We found a prevalence of 3.6 and 3.3% for COPD and asthma respectively. Our algorithm was employed to favour the specificity of detection. In other words, since it is unlikely that an individual who does not suffer from COPD (or asthma) is hospitalized with a diagnosis of COPD (or asthma), and/or uses a medication to treat COPD (or asthma) and/or benefits of exemption for asthma, the rate of false positives detected by our algorithm is expected to be close to zero. It is not surprising that the prevalence estimates obtained by other algorithms, mainly based on drug dispensation [9, 10], widely disagree with ours, likely due to 1) false positive reports (e.g. of patients suffering of bronchitis and bronchiectasis) and/or 2) too broad drug categories (e.g. any respiratory medicament) [6, 16, 17].

However, we realized that, despite the high expected specificity, our algorithm is affected by no optimal sensitivity, being the latter a very serious weakness for investigations aimed to measure the burden of disease. In fact, we found that just over half and one third of patients suffering from COPD and asthma were respectively detected from our algorithm, making prevalence seriously biased towards underestimation. However, measuring sensitivity of our algorithm through an external validation, adjusted prevalence of 6.8% (COPD among person aged 40 years or older) and 8.5% (asthma among person aged 40 years or younger) were obtained. These figures should be compared with the 8.8% prevalence of COPD in adults aged ≥40 years and 7.4% prevalence of asthma in persons aged <44 years, reported for the 28 countries of the European Union around 2010 [25]. The risk of misdiagnosis of COPD and asthma in general practice is generally considered to be of some concern [18] and for this reason GPs’ reports must to be considered an imperfect gold-standard for validation of our algorithms. It follows that the prevalence of COPD and asthma obtained in our study should be considered biased, even if they were corrected for potential misclassification of the diagnosis. It should be emphasized, however, that our prevalence estimates were similar to those obtained assuming a sensitivity of GPs diagnosis of 0.77 and 0.81 for COPD and asthma respectively (i.e., those found in a recent Multicentric Italian study [26]) and specificity close to 1 (i.e., with values of 0.98 and 1.00 for COPD and asthma respectively).

The raw prevalence estimates showed in the present study seems to be lower compared to those reported in other studies regarding the Italian population available in the scientific literature. Different reasons may explain these observed differences; first of all, a lack of homogeneity in the criteria used to identify COPD and asthma cases is observed. In fact, every study uses a different algorithm for case definition characterized by its own predictive value affecting the number of cases detected and consequently the prevalence estimates. Secondly, the sources of data (HCUs, surveys, GPs) used to estimate prevalence in the scientific literature are characterized by different level of completeness and information that may influence the prevalence estimates obtained. In particular, the surveys can include patients who may have mild symptoms that do not lead them to seek medical care and often investigate unspecific symptoms rather than carefully diagnosed diseases.

It must be mentioned that HCUs are a useful data source to investigate the prevalence of diseases because they describe in a very accurate way the real world practice but on the other hand, they cannot capture patients affected by COPD or asthma who do not require specific care, not access to healthcare [27] or not consult the GPs [28].

## Conclusions

In conclusion, our study confirms and adds further evidence that COPD and asthma should be considered important public health issues also in Italy since almost 9% of children and young adults and 7% of older adults is actually affected by asthma or COPD respectively. As a novel and original message, our study showed that HCU databases are useful sources for estimating prevalence of COPD and asthma, provided that validated algorithms combining the use of several healthcare service are applied for detecting ill patients. This is of great importance because, given the wide availability of high quality HCU data, monitoring and comparing burden of chronic respiratory diseases, as well as evaluating the impact of public health services, is easily accomplished with limited efforts.

## Additional file

Additional file 1:
**Table S1.** ICD-9 CM, ATC and exemption codes used in the reference algorithm and comparison algorithms applied to capture COPD and asthma cases among the beneficiaries of the Regional Health Service. Lombardy, Italy. (DOCX 15 kb)

## Abbreviations

COPD: Chronic obstructive pulmonary disease
CRDs: Chronic respiratory diseases
GPs: General practitioners
HCU: Healthcare utilization databases
ICS: Inhaled corticosteroids
LABAs: Long-acting beta agonists
NHS: National health service
prescr/yr.: Prescription per year
SABAs: Short-acting beta agonists
SE: Sensitivity
