# Supplementary material for: Can healthcare utilization data reliably capture cases of chronic respiratory diseases? a cross-sectional investigation in Italy
Source: BMC Pulm Med. 2017 Jan 19;17:20. doi: 10.1186/s12890-016-0362-6 (PMC5248488; doi:10.1186/s12890-016-0362-6)
Supplement: Additional file 1: — Table S1. ICD-9 CM, ATC and exemption codes used in the reference algorithm and comparison algorithms applied to capture COPD and asthma cases among the beneficiaries of the Regional Health Service. Lombardy, Italy. (DOCX 15 kb) [file 12890_2016_362_MOESM1_ESM.docx]

**Supplementary Table 1**. ICD-9 CM, ATC and exemption codes used in the reference algorithm and comparison algorithms applied to capture COPD and asthma cases among the beneficiaries of the Regional Health Service. Lombardy, Italy

|  | **Class** | **Drug** | **ATC** |
| --- | --- | --- | --- |
| **Prescription database** | LABA (long acting beta adrenergic) | Salmeterol^*, **^ | R03AC12 |
|  |  | Formoterol^*, **^ | R03AC13 |
|  |  | Indacaterol^**^ | R03AC18 |
|  | LABA/ICS fixed combination | Formoterol/Budesonide^*, **^ | R03AK07 |
|  |  | Salmeterol/Fluticasone^*, **^ | R03AK06 |
|  | SABA (short acting β2 adrenergic) | Salbutamol^*, **^ | R03AC02 |
|  |  |  | R03CC02 |
|  |  | Fenoterol | R03AC04*^,^ ** |
|  |  |  | R03CC04* |
|  |  | Terbutaline^**^ | R03AC03 |
|  | ICS (inhaled corticosteroid) | Beclometasone^*, **^ | R03BA01 |
|  |  | Fluticasone^*, **^ | R03BA05 |
|  |  | Flunisolide^*, **^ | R03BA03 |
|  |  | Budesonide^*, **^ | R03BA02 |
|  |  | Ciclesonide^**^ | R03BA08 |
|  | Chromones | Cromoglicic acid^*^ | R03BC01 |
|  |  | Nedocromil^*^ | R03BC03 |
|  | Anticholinergics, SAMA (Short acting muscarinic antagonist) | Ipratropium bromide^*, **^ | R03BB01 |
|  |  | Oxitropium bromide^*, **^ | R03BB02 |
|  | Anticholinergics, LAMA (Long acting muscarinic antagonist) | Tiotropium bromide^**^ | R03BB04 |
|  | SABA/SAMA | Salbutamol/Ipratropium bromide^**^ | R03AL02 |
|  |  | Fenoterol/Ipratropium bromide^**^ | R03AL01 |
|  | Xanthines | Theophylline^*^ | R03DA04 |
|  | LTRA (leukotriene receptor antagonist) | Zaphirlukast^*^ | R03DC01 |
|  |  | Montelukast^*^ | R03DC03 |
|  | Corticosteroids for systemic use^*^ |  | H02 |
|  | Antibacterial for systemic use^*^ |  | J01 |
| **Exemptions database** |  | | **Code** |
|  | Exemption of asthma | | 007.493 |
| **Hospital discharge database** |  | | **ICD-9 CM codes** |
|  | COPD^x^ | | 491.XX |
|  |  |  | 492.XX |
|  |  |  | 496.XX |
|  | Asthma^x^ | | 493.XX |

^x^ All codes belonging the ICD-9 category

^*^ Asthma only; ^**^COPD only
